# The Impact of COVID-19 on Distress Tolerance in Pakistani Men and Women

**DOI:** 10.3389/fpsyg.2022.852121

**Published:** 2022-06-07

**Authors:** Salman Shahzad, Wendy Kliewer, Nasreen Bano, Nasreen Begum, Zulfiqar Ali

**Affiliations:** ^1^Institute of Clinical Psychology, University of Karachi, Karachi, Pakistan; ^2^Department of Psychology, Virginia Commonwealth University, Richmond, VA, United States; ^3^Department of Applied Psychology, Virtual University of Pakistan, Lahore, Pakistan

**Keywords:** sex differences, COVID-19, distress tolerance, well-being, Pakistan

## Abstract

The novel coronavirus (COVID-19) is an infectious disease that spread across the world, bringing with it serious mental health problems for men and women. Women in Pakistan are infected with COVID-19 at a much lower rate than men, yet report worse mental health. To explain this paradox, we surveyed 190 participants (46% male) shortly following the country lockdown, focusing on perceptions of the COVID-19 impact and positive adjustment. Measures used in this study included the Warwick-Edinburgh Mental Well-being Scale and Distress Tolerance Scale. Factor analysis revealed five distinct areas related to COVID-19, which did not differ by sex. However, men reported higher levels of both distress tolerance and well-being than women. High endorsement of actions to protect against COVID-19 was related to lower distress tolerance scores, but in different ways for men and women. Men, but not women, who endorsed more protective measures to stop the pandemic reported higher DTS absorption scores, and therefore being more consumed by distress; women who endorsed more protective measures to stop the pandemic reported less acceptance of distress than men, as reflected in DTS appraisal scores. An in-depth analysis of women’s beliefs and behaviors related to COVID-19 is warranted to understand why Pakistani women who are infected with COVID-19 at lower rates than men show more mental health symptoms.

## Introduction

The COVID-19 pandemic had a devastating effect on people’s lives. With its emergence, it has shaken the global economy including Pakistan, and it changed the country’s social fabric and the healthcare system. When the first case of COVID-19 was reported in Pakistan on 26 February 2020 (Waris et al., 2020b), people were shocked, fearful, and felt insecure. This largely was the case because the nation was not prepared for such a crisis, though the Government of Pakistan took various measures to reduce the risk of contracting COVID-19, such as wearing face masks, maintaining social distancing, not shaking hands, and washing one’s face and hands. Further, the Government initiated a lockdown across the country in early March 2020. At that time, self-quarantine and social distancing were the only available options to reduce the spread of COVID-19 (Harper et al., 2020). As a result, the Pakistani population faced many challenges including increases in physical and mental health problems.

The mental health consequences resulting from COVID-19 have been widely observed (Lima et al., 2020). Panic, fear, a sense of insecurity, and other stress responses were observed worldwide. Lockdowns imposed in early 2020 to control the spread of the virus pushed people to stay in their homes for long periods of time. This isolation led to serious mental health issues including anxiety, depression, frustration, irritability, insomnia, post-traumatic stress symptoms, and aggression (Brooks et al., 2020; Di Giuseppe et al., 2020; Franceschini et al., 2020; Salari et al., 2020). In a systematic review that included data from China, Spain, Italy, Iran, the United States, Turkey, Nepal, and Denmark, Xiong et al. (2020) found a high prevalence of depression, anxiety, post-traumatic stress disorder (PTSD), and stress among the general population during the COVID-19 pandemic. For example, a study conducted in China found that 35% of participants reported distress, including 2.9% who reported PTSD (Qiu et al., 2020). Additionally, Tang et al. (2020) found that 9% of Chinese University students who were home quarantined reported clinical levels of depressive symptoms. In a study conducted in the United States and Israel, 22.2% of the sample reported having generalized anxiety, while 16.1% reported having depressive symptoms (Barzilay et al., 2020). During the first week of the lockdown, 20% of participants in a study from Italy reported experiencing mental health issues, such as depression, anxiety, and stress; 37% of the sample reported having symptoms related to post-traumatic stress (Rossi et al., 2020). In general, the literature on responses to COVID-19 did not include reports of positive adjustment.

Among the psychological dimensions that mediate the relationship between stressors and mental health outcomes in the face of lockdowns, researchers have found that coping strategies and the ability to bounce back (i.e., resilience) matter (Serafini et al., 2020). For example, in a study conducted during the acute COVID-19 outbreak, Barzilay et al. (2020) found that participants who reported higher levels of resilience evidenced fewer mental health problems, such as depression or anxiety. These findings were supported by Morales-Vives et al. (2020) who found that individuals who adjusted well to the lockdown had higher levels of resilience and better coping strategies. As noted by Fletcher and Sarkar (2013), individuals who have the ability to bounce back evaluate stressors differently than those who do not, and this evaluation leads to the use of more effective coping strategies to deal with stressors. However, we could locate no research on responses to the pandemic that examined how associations between beliefs and behaviors associated with the pandemic (that is, appraisals and coping efforts) and mental health differed for men and women.

Similar to many countries in the world, Pakistani citizens at large have been negatively affected by the COVID-19 pandemic and government interventions to manage the spread of the virus (Khan et al., 2021). Despite the fact that the female COVID-19 infection rate is just over half of the infection rate of males in Pakistan (Waris et al., 2020a), studies both in Pakistan (Khan et al., 2021), and other Asian countries (Othman, 2020; Qiu et al., 2020), as well as in the middle east (Abufaraj et al., 2021) reported that women demonstrated worse mental health in response to the pandemic than men. The present study aimed to understand this apparent contradiction by identifying concerns related to the COVID-19 pandemic, sex differences in these concerns, and associations of these concerns with mental well-being across a broad spectrum of people in Pakistan. Importantly, the current study goes beyond merely examining sex differences in adjustment, and considers how concerns related to the pandemic may be differentially associated with adjustment for men versus women. This has not been evaluated in prior studies. Further, the current study focuses on positive aspects of adjustment. In sum, our research questions were: (1) In the 3-month period immediately following the lockdown, what beliefs about and responses to the COVID-19 pandemic were expressed by men and women living in Pakistan? (2) To what extent were beliefs about and responses to the COVID-19 pandemic associated with well-being? (3) Did the patterns of association between beliefs about and responses to the COVID-19 pandemic and well-being differ for males and females?

## Methods

### Participants

The Ethics Committee of the Institute of Clinical Psychology, University of Karachi, approved the study. Participants (*N* = 190; 46% male; *M* age = 28.22, *SD* = 6.68 years) were recruited using social media platforms (WhatsApp, Facebook, messenger, and email) throughout Pakistan using the snowball sampling technique. Anonymous data were collected using Google Forms between 20 March and 30 June 2020, a period immediately following the lockdown due to the COVID-19 pandemic. Table 1 provides demographic information on the sample.

**Table 1:** Demographic characteristics of study participants.

Demographic variable	Total sample *N* (%)	Males *N* (%)	Females *N* (%)
Number	190	88 (46.3)	102 (53.7)
Age (years) Mean (±*SD*)	28.22 (6.68)	29.95 (7.92)	26.72 (4.92)
Family Size Mean (±*SD*)	6.05 (2.48)	6.30 (2.58)	5.84 (2.38)
**Marital status**			
Single	134 (70.5)	54 (61.4)	80 (78.4)
Married	53 (27.9)	34 (38.6)	19 (18.6)
Divorced	3 (1.6)	0	3 (2.9)
**Socioeconomic level**			
Lower	2 (1.1)	2 (2.3)	0
Lower middle	22 (11.6)	15 (17.0)	7 (6.9)
Middle	158 (83.2)	69 (78.4)	89 (87.3)
Upper	8 (4.2)	2 (2.3)	6 (5.9)
**Educational attainment**			
Grade 12	4 (2.1)	3 (3.4)	1 (1.0)
Grade 14	60 (31.6)	28 (31.8)	32 (31.4)
Grade 15	2 (1.1)	0	2 (2.0)
Grade 16	67 (35.3)	35 (39.8)	32 (31.4)
Above Grade 16	57 (30.0)	22 (25.0)	35 (34.3)
**Occupation**			
Student	55 (28.9)	23 (26.1)	32 (31.4)
Other professional	135 (71.1)	65 (73.9)	70 (68.6)
**Province**			
Sindh	106 (55.8)	37 (42.0)	69 (67.6)
Gilgit Baltistan	50 (26.3)	32 (36.4)	18 (17.6)
KPK	11 (5.8)	7 (8.0)	4 (3.9)
Balochistan	2 (1.1)	1 (1.1)	1 (1.0)
Punjab	20 (10.5)	10 (11.4)	10 (9.8)
AJK	1 (0.5)	1 (1.1)	0

### Measures

### Participants Self-Reported All Measures in English

#### Demographic Information

Participants reported their age, biological sex (female or male), number of family members living together at the time of the survey, marital status (married, single, divorced), socioeconomic status (SES), educational attainment, profession, the province in which they lived, and whether they had a mental health diagnosis or any symptoms of COVID-19.

#### Questions on COVID-19

At the time the data were collected, no other measures specifically designed to assess appraisals and coping efforts associated with the pandemic had been developed. Based on clinical observations, as well as an appraisal and coping efforts theoretical framework (see Lazarus and Folkman, 1984), 13 statements were written regarding beliefs about (appraisals) and responses to (coping efforts) the COVID-19 pandemic. Respondents indicated their agreement with each statement on a five-point Likert scale ranging from 1 (*strongly disagree*) to 5 (*strongly agree*), a format widely used in the psychological literature.

Because this was a new measure, the 13 statements were subjected to an Exploratory Factor Analysis (EFA) with principal components as the extraction method and varimax rotation and Kaiser normalization using SPSS 27. This method maximizes distinctions between factors. Five factors with eigenvalues greater than 1 were extracted, and these explained 62.42 percent of the variance (see Table 2). The factors were: (1) endorsement of protective action (items 2, 12, and 13); (2) emotional impact (items 5, 10, and 11); (3) physical impact (items 4 and 9); (4) adjustment challenges (items 7 and 8); and (5) economic and social impact (items 3 and 6). Item 1 did not clearly load on one factor and was not included in subsequent analyses.

**Table 2:** COVID-19 items and exploratory factor analysis results.

COVID-19 items	Component extracted				
	1	2	3	4	5
1- I worry about the COVID-19 pandemic.	0.328	0.544	0.172	−0.164	−0.402
2- I think it is important to close borders and quarantine every person coming in to stop the pandemic.	**0.753**	0.109	−0.124	0.010	0.144
3- This pandemic affects me economically.	0.256	−0.068	0.323	0.045	**0.637**
4- This pandemic affects me physically.	−0.049	0.190	**0.649**	−0.046	0.045
5- This pandemic affects me emotionally.	0.157	**0.663**	0.391	0.042	0.150
6- This pandemic affects me socially.	0.042	0.385	−0.115	−0.068	**0.698**
7- I have a hard time adjusting to the changes the COVID-19 pandemic has created.	−0.082	0.200	−0.189	**0.692**	0.266
8- I think I am at greater risk to get virus.	0.061	−0.092	0.123	**0.813**	−0.208
9- I am worried about myself during this pandemic.	−0.036	−0.043	**0.709**	0.023	−0.003
10- I am worried about my family, friends, and significant others.	−0.038	**0.671**	−0.103	−0.018	0.070
11- This pandemic has changed my and my family’s lifestyle.	0.290	**0.639**	0.148	0.312	0.162
12- I use safety measures to stop the COVID-19 virus.	**0.891**	0.054	0.101	0.033	0.027
13- I maintain social distancing.	**0.856**	0.116	−0.053	−0.029	0.020

*Factor 1, endorsement of protective action (items 2, 12, and 13); Factor 2, emotional impact (items 5, 10, and 11); Factor 3, physical impact (items 4 and 9); Factor 4, adjustment challenges (items 7 and 8); and Factor 5, economic and social impact (items 3 and 6). Bolded values indicate the factor to which the item was assigned*.

Factor scores were computed by multiplying the factor loadings from the EFA by the items and adding the product terms. The specific computations were as follows: COVID Fac1 = ((covid2 * 0.753) + (covid12 * 0.891) + (covid13 * 0.856)); COVID Fac2 = ((covid5 * 0.663) + (covid10 * 0.671) + (covid11 * 0.639)); COVID Fac3 = ((covid4 * 0.649) + (covid9 * 0.709)); COVID Fac4 = ((covid7 * 0.692) + (covid8 * 0.813)); and COVID Fac5 = ((covid3 * 0.637) + (covid6 * 0.698)). For each factor score, higher values reflected more of the construct. Descriptive information on each factor score is reported for males and females separately in Table 3.

**Table 3:** Descriptive information on and correlations among study constructs by sex.

	1	2	3	4	5	6	7	8	9	*M* females	*SD* females
1—COVID-19 factor 1: Protective action	1	0.28^**^	−0.06	0.03	0.35^***^	0.02	0.05	−0.22^*^	−0.07	10.96	2.02
2—COVID-19 factor 2: Emotional impact	0.30^**^	1	0.15	0.25^*^	0.30^**^	−0.06	−0.22*	0.09	−0.07	7.61	1.55
3—COVID-19 factor 3: Physical impact	0.08	0.33^**^	1	−0.03	0.07	0.03	−0.06	0.23^*^	0.14	5.21	1.07
4—COVID-19 factor 4: Adjustment challenges	−0.01	−0.09	0.02	1	−0.02	−0.14	−0.03	0.24^*^	−0.05	6.20	1.37
5—COVID-19 factor 5: Economic and social impact	0.16	0.41^***^	0.08	0.11	1	−0.03	−0.04	0.03	−0.01	5.29	1.05
6—DTS Tolerance subscale	−0.12	−0.09	−0.07	0	−0.14	1	−0.03	0.06	0.40^***^	5.43	1.89
7—DTS Absorption subscale	−0.27^*^	−0.20	0.10	−0.05	−0.04	0.07	1	−0.02	0.18	4.44	1.01
8—DTS Appraisal subscale	0.06	0.11	0.09	0.05	0.08	0.16	0.12	1	0.39^***^	10.37	2.03
9—Well-being	0	−0.02	−0.18	−0.14	−0.11	0.31^**^	0.18	0.20	1	24.94	5.06
*M* males	10.34	7.54	5.27	6.19	5.55	9.91	9.65	17.50	40.56		
*SD* males	2.44	1.50	0.96	1.05	1.07	1.35	1.51	2.56	6.18		

**N* = 102 females, 88 males. DTS, Distress tolerance scale. Values above the diagonal are for women, and values below the diagonal for men. Possible range on the COVID-19 factors were as follows: Factor 1—2.5 to 12.5; Factor 2—1.97 to 9.87; Factor 3—1.36 to 6.79; Factor 4—1.51 to 7.53; Factor 5—1.34 to 6.68. Possible range on the DTS subscales were as follows: Tolerance—3 to 15; Absorption—3 to 15; Appraisal—6 to 30. Possible range on the well-being scale: 14 to 70*.

* *p < 0.05*,

** *p < 0.01*,

*** *p < 0.001*.

#### Distress Tolerance Scale

The 15-item Distress Tolerance Scale (DTS; Simons and Gaher, 2005) was used to assess participants’ ability to experience and endure negative emotional states. Respondents rate items on a five-point Likert scale ranging from 1 (*strongly agree*) to 5 (*strongly disagree*). In addition to a total score, the DTS includes four subscales related to (1) perceived ability to tolerate emotional distress (Tolerance; 3 items; “Feeling distressed or upset is unbearable to me”), (2) subjective appraisal of distress (Appraisal; 6 items; e.g., “My feelings of distress of being upset are not acceptable”), (3) attention absorbed by negative emotions (Absorption; 3 items; e.g., “When I feel distressed or upset, all I can think about is how bad I feel”), and (4) regulation efforts to alleviate distress (Regulation; 3 items; e.g., “I’ll do anything to stop feeling distressed or upset”). One item on the appraisal subscale is reverse coded. Higher scores on these subscales reflect more tolerance of negative affect (tolerance), not being consumed by negative affect (absorption), greater acceptability of negative affect (appraisal), and more regulation of negative affect (regulation). In a validation study, Simons and Gaher (2005) reported high internal consistency (α = 0.89), adequate 6-month test–retest reliability (*r* = 0.61), and appropriate convergence with other self-report ratings of affective distress and regulation. In the current study, Cronbach alphas were acceptable for Tolerance (α = 0.64), Appraisal (α = 0.73), and Absorption (α = 0.84). The alpha for regulation was low and was not used in the analyses. To compute each subscale items were added. The specific computations were as follows: DTS tolerance = dts1 + dts3 + dts5; DTS absorption = dts2 + dts4 + dts15; DTS appraisal = r_dts6 + dts7  + dts9 + dts10 + dts11 + dts12. Descriptive information on each DTS subscale is reported for males and females separately in Table 3.

#### Mental Well-Being

The 14-item Warwick-Edinburgh Mental Well-being Scale (Tennant et al., 2007) was used to assess positive mental health. Respondents rate each item on a five-point Likert scale ranging from 1 (*none of the time*) to 5 (*all of the time*). A sample item is “I’ve been interested in new things.” Items are summed to create a total score with higher scores indicate better mental well-being. The specific computation was as follows: Well-being = mwb1 + mwb2 + mwb3 + mwb4+ mwb5+ mwb6 + mwb7  + mwb8 + mwb9 + mwb10 + mwb11+ mwb12 + mwb13 + mwb14. This scale has good psychometric properties including acceptable one-week test–retest reliability (*r* = 0.83), internal consistency (α = 0.91 for a population sample), and good discriminant validity (Tennant et al., 2007). Internal consistency in the current study was acceptable (α = 0.86). Descriptive information on this measure is reported separately for males and females in Table 3.

#### Procedure

All participants read the consent form and explicitly agreed to participate in this study on a voluntary basis before starting this online survey. Only those participants who were above age 18 were included in this study. The order of measures in the survey was demographics, questions on COVID-19, the Distress Tolerance Scale, and the Mental Well-Being Scale. Participants were provided the email address of the principal investigator and were asked to email if they need any assistance during completion of the survey. Only participants who completed the survey were included in the analyses. Participants were not compensated for their participation.

### Data Analysis

All analyses were conducted using SPSS 27. Results of the Exploratory Factor Analysis (EFA) are presented in Table 2, followed by descriptive information on and correlations among the study variables in Table 3. Regression analyses predicting the distress tolerance subscales and mental well-being are presented in Table 4. A *value of p* of <0.05 was considered significant.

**Table 4:** Regression analyses predicting well-being and distress tolerance from demographics and COVID-19 factor scores.

Variables	Unstd coefficients		Std coeff	Value of *p*	95% CI for B	
	B	SE	Beta		Lower bound	Upper bound
**Outcome: absorption subscale of distress tolerance scale**						
Constant	4.772	0.293		<0.001	4.194	5.351
Sex	**5.236**	**0.297**	**0.913**	**<0.001**	**4.649**	**5.822**
SES	0.184	0.212	0.028	0.385	−0.233	0.602
Education	−0.051	0.076	−0.023	0.501	−0.201	0.099
Age	−0.022	0.019	−0.052	0.238	−0.059	0.015
Marital Status	−0.502	0.266	−0.079	0.061	−1.027	0.023
COVID-19 factor 1: protective action	0.072	0.066	0.057	0.271	−0.057	0.202
COVID-19 factor 2: emotional impact	**−0.181**	**0.087**	**−0.096**	**0.039**	**−0.354**	**−0.009**
COVID-19 factor 3: physical impact	−0.041	0.114	−0.015	0.720	−0.267	0.185
COVID-19 factor 4: adjustment challenges	0.018	0.092	0.007	0.850	−0.165	0.200
COVID-19 factor 5: economic and social impact	−0.011	0.125	−0.004	0.929	−0.259	0.236
COVID-19 factor 1 × sex	**−0.206**	**0.085**	**−0.120**	**0.016**	**−0.374**	**−0.038**
COVID-19 factor 2 × sex	−0.110	0.137	−0.039	0.422	−0.380	0.160
COVID-19 factor 3 × sex	0.330	0.184	0.075	0.075	−0.033	0.694
COVID-19 factor 4 × sex	−0.206	0.156	−0.051	0.187	−0.514	0.101
COVID-19 factor 5 × sex	0.110	0.182	0.035	0.545	−0.248	0.468
**Outcome: appraisal subscale of distress tolerance scale**						
Constant	10.669	0.530		<0.001	9.623	11.715
Sex	**6.771**	**0.546**	**0.809**	**<0.001**	**5.694**	**7.848**
SES	0.116	0.390	0.012	0.766	−0.653	0.885
Education	0.014	0.138	0.004	0.918	−0.259	0.288
Age	−0.062	0.034	−0.099	0.071	−0.130	0.005
Marital status	0.080	0.483	0.009	0.869	−0.874	1.033
COVID-19 factor 1: protective action	**−0.292**	**0.118**	**−0.151**	**0.015**	**−0.525**	**−0.058**
COVID-19 factor 2: emotional impact	−0.007	0.158	−0.002	0.966	−0.318	0.305
COVID-19 factor 3: physical impact	0.379	0.207	0.092	0.069	−0.029	0.787
COVID-19 factor 4: adjustment challenges	**0.370**	**0.167**	**0.107**	**0.028**	**0.040**	**0.700**
COVID-19 factor 5: economic and social impact	0.268	0.227	0.068	0.239	−0.180	0.715
COVID-19 factor 1 × sex	**0.461**	**0.161**	**0.170**	**0.005**	**0.142**	**0.779**
COVID-19 factor 2 × sex	0.068	0.255	0.016	0.792	−0.436	0.571
COVID-19 factor 3 × sex	−0.246	0.341	−0.038	0.471	−0.919	0.427
COVID-19 factor 4 × sex	−0.268	0.296	−0.043	0.367	−0.852	0.316
COVID-19 factor 5 × sex	−0.382	0.339	−0.083	0.262	−1.051	0.288
**Outcome: tolerance subscale of distress tolerance scale**						
Constant	4.714	0.390		<0.001	3.943	5.484
Sex	**4.503**	**0.393**	**0.810**	**<0.001**	**3.728**	**5.279**
SES	−0.016	0.283	−0.002	0.955	−0.575	0.543
Education	**−0.245**	**0.101**	**−0.111**	**0.017**	**−0.444**	**−0.045**
Age	0.030	0.025	0.072	0.240	−0.020	0.080
Marital Status	**0.725**	**0.356**	**0.118**	**0.044**	**0.021**	**1.428**
COVID-19 factor 1: protective action	0.017	0.087	0.014	0.842	−0.154	0.189
COVID-19 factor 2: emotional impact	0.040	0.117	0.022	0.731	−0.190	0.270
COVID-19 factor 3: physical impact	0.066	0.152	0.024	0.662	−0.233	0.366
COVID-19 factor 4: adjustment challenges	0.025	0.131	0.010	0.850	−0.234	0.284
COVID-19 factor 5: economic and social impact	−0.058	0.166	−0.023	0.726	−0.387	0.270
COVID-19 factor 1 × sex	−0.059	0.113	−0.035	0.603	−0.282	0.164
COVID-19 factor 2 × sex	−0.072	0.181	−0.026	0.692	−0.429	0.285
COVID-19 factor 3 × sex	−0.102	0.244	−0.024	0.677	−0.583	0.380
COVID-19 factor 4 × sex	0.040	0.211	0.010	0.851	−0.376	0.455
COVID-19 factor 5 × sex	−0.084	0.241	−0.028	0.728	−0.559	0.391
**Outcome: well-being total**						
Constant	24.287	1.246		<0.001	21.828	26.746
Sex	**15.509**	**1.258**	**0.845**	**<0.001**	**13.026**	**17.993**
SES	−0.427	0.957	−0.019	0.656	−2.316	1.463
Education	0.124	0.323	0.017	0.701	−0.513	0.762
Age	−0.072	0.080	−0.053	0.369	−0.231	0.086
Marital Status	0.622	1.130	0.031	0.582	−1.608	2.852
COVID-19 factor 1: protective action	−0.195	0.276	−0.047	0.481	−0.740	0.350
COVID-19 factor 2: emotional impact	−0.250	0.369	−0.042	0.498	−0.978	0.477
COVID-19 factor 3: physical impact	0.817	0.482	0.090	0.092	−0.135	1.770
COVID-19 factor 4: adjustment challenges	−0.089	0.391	−0.012	0.821	−0.860	0.683
COVID-19 factor 5: economic and social impact	0.051	0.531	0.006	0.924	−0.997	1.099
COVID-19 factor 1 × sex	0.323	0.362	0.058	0.372	−0.390	1.037
COVID-19 factor 2 × sex	0.306	0.574	0.034	0.595	−0.827	1.438
COVID-19 factor 3 × sex	−1.149	0.795	−0.080	0.150	−2.718	0.420
COVID-19 factor 4 × sex	−0.856	0.656	−0.067	0.194	−2.150	0.439
COVID-19 factor 5 × sex	−0.132	0.774	−0.013	0.865	−1.659	1.396

*CI, confidence interval. Sex was coded 0 = female, 1 = male. Marital status was coded 0 = married, 1 = single, including divorced. Overall model fit indices were as follows: Outcome DTS absorption, *F*(15, 173) = 60.83, *p* < 0.001; Adjusted R^2^ = 0.827.Outcome DTS appraisal, *F*(15, 171) = 35.18, *p* < 0.001; Adjusted R^2^ = 0.734. Outcome DTS tolerance, *F*(15, 172) = 27.11, *p* < 0.001; Adjusted R^2^ = 0.680.Outcome Well-being, *F*(15, 171) = 29.91, *p* < 0.001; Adjusted R^2^ = 0.700. Significant terms in the models are bolded*.

## Results

### Descriptive Information on and Correlations Among Study Variables

Table 3 presents descriptive information on the key study variables and correlations among separately for males and females. Results of *t*-tests and chi-square analyses, as appropriate, indicated that the COVID-19 factors were not significantly associated with age, family size, marital status, socioeconomic level, educational attainment, or occupation. Further, there were no significant sex differences on any of the COVID-19 factors: COVID-19 factor 1: *t*(188) = 1.93, *p* = 0.055; COVID-19 factor 2: *t*(188) = 0.31, *p* = 0.78; COVID-19 factor 3: *t*(188) = −0.41, *p* = 0.68; COVID-19 factor 4: *t*(188) = 0.02, *p* = 0.98; COVID-19 factor 5: *t*(188) = −1.69, *p* = 0.092. However, males reported higher levels of the distress tolerance and well-being than females, DTS—Tolerance: *t*(188) = −18.98, *p* < 0.001; DTS—Absorption: *t*(188) = −27.49, *p* < 0.001; DTS—Appraisal: *t*(188) = −21.06, *p* < 0.001; Well-being: *t*(188) = −18.87.

### Regression Analyses Predicting Well-Being and Distress Tolerance Subscales

A series of linear regression analyses were conducted to evaluate the contribution of COVID-19 factors on distress tolerance subscales and well-being, after accounting for demographics. Interactions of COVID-19 factors with biological sex were included in each model to determine whether patterns of association differed for males and females. All continuous level covariates and predictors were centered, and interaction terms were computed using the centered variables. Each regression included the demographic covariates, the five COVID-19 factors, and the interactions of the five COVID-19 factors with sex. All variables were entered simultaneously. Multivariate outliers were assessed with Cook’s D distance measure (Cook and Weisberg, 1982) and removed when indicated (see Table 4).

**Figure 1:**
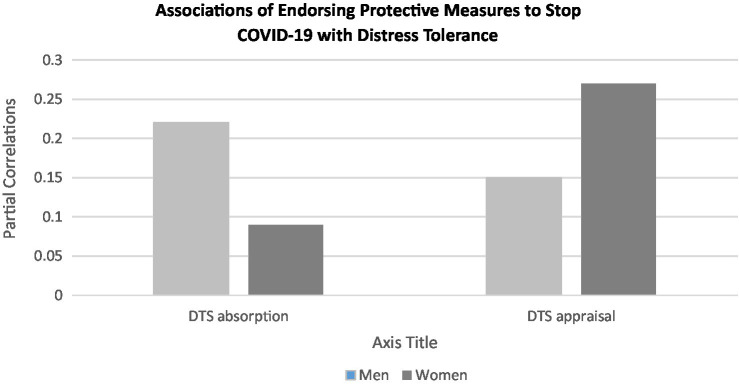
Associations of endorsing protective measures to stop COVID-19 (Factor 1) with distress tolerance subscales for males and females.

Analyses revealed a main effect of COVID-19 factor 2: emotional impact on DTS Absorption, a main effect of COVID-19 factor 4: adjustment challenges on DTS Appraisal, a main effect of COVID-19 factor 1: protective action on DTS Appraisal, and interactions of COVID-19 factor 1 with sex on both DTS Absorption and Appraisal. Participants who reported a greater emotional impact of COVID-19 (factor 2) also reported being more focused on and consumed by distress, but participants who reported more adjustment challenges (factor 4) indicated they were more accepting of distress. Participants who endorsed more protective measures to stop the pandemic (factor 1) were less accepting of negative emotions (DTS appraisal), but the strength of this association differed by sex, with women evidencing a stronger association than men. Additionally, men, but not women, who endorsed more protective measures to stop the pandemic reported being more consumed by distress, as reflected in DTS absorption scores (see Figure 1). In contrast, in the models predicting either the DTS tolerance subscale or well-being there were no main effects of COVID-19 factors nor any interactions with sex.

## Discussion

This study investigated beliefs and behaviors around COVID-19 and their association with mental health in men and women living in Pakistan in order to understand sex differences in adjustment to the pandemic. Importantly, this study extended prior literature by examining sex differences in patterns of associations between COVID-19-related beliefs and behaviors and mental health and well-being. The study yielded five unique findings: behaviors and beliefs related to COVID-19 fell into five distinct categories and did not differ by sex; both men and women who indicated greater emotional impact of COVID-19 also reported being more focused on and consumed by negative affect; both men and women who reported more adjustment challenges to COVID-19 reported greater acceptance of distress; participants who endorsed more protective measures to stop the pandemic were less accepting of negative emotions, with women evidencing stronger associations than men; and finally, men, but not women, who endorsed more protective measures to stop the pandemic reported being more consumed by distress.

First, it is probably not surprising that multiple categories of COVID-19 beliefs and behaviors emerged from the analyses. Emotional, physical, and economic and social impact each formed distinct factors, which also were differentiated from adjustment challenges and from coping behaviors, represented by endorsement of protective action items. Individuals evidence diverse responses to life stressors, and cognitive and behavioral responses are two types of responses seen in most coping inventories.

Second, both men and women reported similar associations to distress tolerance for two of the COVID-19 factors: emotional impact and adjustment challenges. The positive association between COVID-19’s emotional impact a focus on negative affect makes sense, given that a focus on emotion is common to both constructs. The findings that men and women who reported more COVID-19 adjustment challenges also were more accepting and tolerant of negative affect generally, while seemingly counterintuitive, illustrates the distinctions between cognitive and affective assessments of COVID-19 impacts. Notably, the Adjustment Challenges factor identified in our study was uncorrelated with any of the other four factors we identified.

Third, the sex differences in these data specifically the stronger association for women than for men of protective measures endorsement and less acceptance of negative emotions may indicate a greater overall effect of the pandemic on women, versus men. This sex difference in the overall impact of COVID-19 was supported by Khan et al.’s (2021) study in Pakistan. The differential response by men and women to the endorsement of protective measures also may be explained in part by known sex differences in coping with trauma, including lower levels of well-being in women versus men (Hu et al., 2017).

Finally, the finding that men but not women showed an association between endorsing protective measures and being consumed by distress may indicate a tendency for men to engage in more active coping and problem-solving relative to women (Carver et al., 1989).

### Study Strengths and Limitations

This study provides a novel contribution to the literature by examining sex differences in the linkages between COVID-19-related beliefs and behaviors and adjustment. The study examined responses in the period immediately following the lockdown, drew participants from across Pakistan, and included assessments of positive well-being as part of the survey protocol. Although novel in several ways, this study had a few limitations. Participation was limited to individuals with an internet connection who could respond to a survey in English; thus, the findings may not generalize to the entire Pakistani population. Further, the cross-sectional design precluded the ability to determine temporal associations between the study constructs. Additionally, the overall sample size is lower than desired, and more women than men completed the survey. Lastly, the questions on COVID-19 were developed specifically for this study, as there were no alternative measures were available at that time. Thus, psychometric data on the measure were not available.

### Recommendations for Future Research and Clinical Practice

In addition to collecting additional data to validate the COVID-19 measure used in the study, future research might use a mixed methods approach to understand more deeply the experience of women in the pandemic. In terms of clinical practice, for future pandemics, having a workforce trained to employ Psychological First Aid as mental health challenges emerge likely would contribute to a reduction in psychological distress in the population.

## Data Availability Statement

The raw data supporting the conclusions of this article will be made available by the authors, without undue reservation.

## Ethics Statement

The study was reviewed and approved the Ethics Committee of the Institute of Clinical Psychology, University of Karachi approved the study. Informed consent was obtained from all participants in the study. The patients/participants provided their written informed consent to participate in this study.

## Author Contributions

SS conceptualized the study, wrote portions of the manuscript, and collected data. WK conducted statistical analyses, wrote portions of the manuscript, and edited the manuscript. NBa write up and improvements in introduction, discussion and references. NBe prepared documents, collected data, and wrote portions of the discussion. ZA collected data. All authors contributed to the article and approved the submitted version.

## Conflict of Interest

The authors declare that the research was conducted in the absence of any commercial or financial relationships that could be construed as a potential conflict of interest.

## Publisher’s Note

All claims expressed in this article are solely those of the authors and do not necessarily represent those of their affiliated organizations, or those of the publisher, the editors and the reviewers. Any product that may be evaluated in this article, or claim that may be made by its manufacturer, is not guaranteed or endorsed by the publisher.

## Abbreviations

DTS: Distress tolerance scale
